# The Effect of Information and Communication Technology and Social Networking Site Use on Older People’s Well-Being in Relation to Loneliness: Review of Experimental Studies

**DOI:** 10.2196/23588

**Published:** 2021-03-01

**Authors:** Georgia Casanova, Daniele Zaccaria, Elena Rolandi, Antonio Guaita

**Affiliations:** 1 Centre for Socio-Economic Research on Ageing National Institute of Health & Science on Ageing Istituto di Ricovero e Cura a Carattere Scientifico Ancona Italy; 2 Centre of Competence on Ageing Department of Business Economics, Health and Social Care University of Applied Sciences and Arts of Southern Switzerland Manno Switzerland; 3 Golgi Cenci Foundation Abbiategrasso Italy

**Keywords:** review, aging, loneliness, older people’s well-being, ICTs, social network sites

## Abstract

**Background:**

In the last decades, the relationship between social networking sites (SNSs) and older people’s loneliness is gaining specific relevance. Studies in this field are often based on qualitative methods to study in-depth self-perceived issues, including loneliness and well-being, or quantitative surveys to report the links between information and communication technologies (ICTs) and older people’s well-being or loneliness. However, these nonexperimental methods are unable to deeply analyze the causal relationship. Moreover, the research on older people’s SNS use is still scant, especially regarding its impact on health and well-being. In recent years, the existing review studies have separately focused their attention on loneliness and social isolation of older people or on the use of ICTs and SNSs in elderly populations without addressing the relationship between the former and the latter. This thorough qualitative review provides an analysis of research performed using an experimental or quasi-experimental design that investigates the causal effect of ICT and SNS use on elderly people’s well-being related to loneliness.

**Objective:**

The aims of this review are to contrast and compare research designs (sampling and recruitment, evaluation tools, interventions) and the findings of these studies and highlight their limitations.

**Methods:**

Using an approach that integrates the methodological framework for scoping studies and the Preferred Reporting Items for Systematic Reviews and Meta-Analyses guidelines for systematic reviews, we identified 11 articles that met our inclusion criteria. A thematic and content analysis was performed based on the ex post categorization of the data on the selected studies, and the data were summarized in tables.

**Results:**

The analysis of the selected articles showed that: (1) ICT use is positively but weakly related to the different measures of older people’s well-being and loneliness, (2) overall, the studies under review lack a sound experimental design, (3) the main limitations of these studies lie in the lack of rigor in the sampling method and in the recruitment strategy.

**Conclusions:**

The analysis of the reviewed studies confirms the existence of a beneficial effect of ICT use on the well-being of older people in terms of reduced loneliness. However, the causal relationship is often found to be weak. This review highlights the need to study these issues further with adequate methodological rigor.

## Introduction

The percentage of people aged 65 years and older in the world will rise to 24% in 2030 [1]. Literature underlines how loneliness is one of the main risk factors that have negative effects on seniors’ health [2]. Loneliness is usually defined as an undesirable subjective experience related to unfulfilled intimate and social needs [3]. In Europe, between 10% and 20% of elderly people in Western and Northern Europe and 30% to 55% in Eastern Europe declared feeling lonely [4]. Many interventions have been adopted to reduce loneliness and increase the well-being of elders. Among them, information and communication technologies (ICTs) have been used to help older adults cope with loneliness [5]. The use of ICTs has grown significantly since the 2000s [6]. ICTs are those technologies that can be used to interlink information technology devices such as PCs with communication technologies such as telephones and their telecommunication networks. Michiels and Van Crowder [7] reported almost 20 years ago how ICTs are an expanding assembly of technologies. In the last decades, PCs, laptops, smartphones, tablets with email, and the internet are examples of ICTs able to connect people and support their social life [8]. These types of ICTs, intended to alleviate loneliness and social isolation among older people, are considered significant in expanding and sustaining social contact and improving emotional well-being [9] and are the focus of this review. Recently, experts’ attention has also been drawn to the role of technologies aimed at promoting social relationships, such as social networking sites (SNSs) [10]. Boyd et al [11] defined the SNSs as web-based services that allow individuals to (1) construct a public or semipublic profile within a bounded system, (2) articulate a list of other users with whom they share a connection, and (3) view and traverse their list of connections and those made by others within the system. At the moment, Facebook, Instagram, LinkedIn, and Twitter are the most popular SNSs. In particular, Facebook seems to be the most used by older people [12]. The relationship between SNSs and loneliness in older people is gaining specific relevance. However, research on older people’s SNS use is still scant, especially regarding their impact on health and well-being. Eggermont and colleagues [13] report that older people consider SNSs useful tools to contrast loneliness, one that should integrate (but not replace) face-to-face contacts. Gibson et al [14] have found that older adults are concerned about privacy issues. Some studies also examine older people’s characteristics that favor SNS adoption. For example, Liu et al [15] report that, in the United States, elderly users of SNSs are more likely to be younger, female, and widowed. Studies in this field are often based on qualitative methods, because they allow studying in-depth self-perceived issues, including loneliness and well-being, but to a lesser extent the causal relationship between them [16]. Among quantitative studies, the survey analysis is most used to report the links between ICTs and older people’s well-being [17] or loneliness [18]. Despite the increased attention gained by these studies, the survey—often applied to cross-sectional analysis—does not allow the detection of a causal relationship. In 2013, Nef et al [12] underlined a shortage of experimental research on the relationship between ICT use and older adult well-being, considered more adequate to analyze the causal relationship between phenomena [19].

In recent years, review studies have focused on loneliness and social isolation of older people [20,21] or on the use of ICTs and SNSs in elderly populations without tackling the relationship between the former issue and the latter [12,22]. One of the few literature reviews focused on the relationship between ICT use and loneliness in older people underlines how this research field involves different theoretical frames from various scientific fields [22]. The existing quantitative experimental studies on these issues have significant limitations that may hamper the relevance of the research findings, such as small and not representative samples [23].

The purpose of this review is to contribute to the literature debate on the effect of SNS use on older people’s well-being with specific attention on loneliness, focusing on experimental and quasi-experimental studies. The main aims are to examine and compare the selected studies, analyzing their protocols (sampling, evaluation tools, and treatments) and their findings to identify strengths and limitations and support the development of further similar studies. According to the aims, we present a thorough qualitative review of experimental studies in this field.

## Methods

### Review Procedures

**Figure 1:**
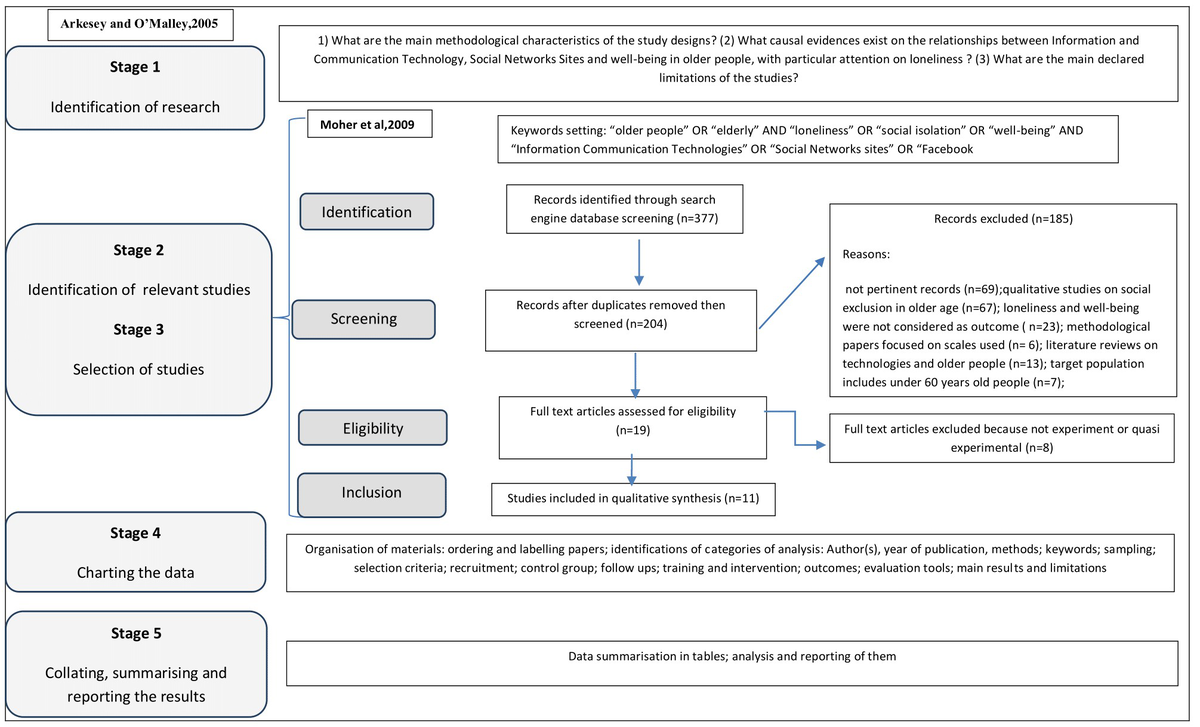
Flowchart of scoping review: methods and procedure.

To ensure a high quality of reporting, this study used the 5 stages for reviews proposed by Arksey and O’Malley [24]: (1) identification of research questions; (2) identification of relevant studies; (3) selection of studies; (4) charting the data; and (5) collating, summarizing, and reporting the results. Taking into account the low spread of the analyzed studies, we decided to improve the method path by the integration of stages 2 and 3 with the 4 Preferred Reporting Items for Systematic Reviews and Meta-Analyses (PRISMA) stages: identification, screening, eligibility, and inclusion [25]. Figure 1 shows the complete method implemented in this review. The combination of these methods ensures the review will stay linear and focused, as proposed by Arksey and O’Malley [24], and limits losing useful papers on the topic thanks to PRISMA approach.

### Identification of Research Questions and Selection Process

In the first stage, we identified 3 research questions: (1) What are the main methodological characteristics of the study designs? (2) What causal evidence exists on the relationships between ICTs, SNSs, and well-being in older people, particularly as regards loneliness? (3) What are the main studies’ declared limitations?

In March 2020 for stages 2 and 3, we performed a comprehensive literature search in the main search engines used in health and social sciences: Scopus, PubMed, Web of Science, and Sociological Abstracts. The search was based on a set of keywords (“older people,” “elderly,” “loneliness,” “well-being,” “information and communication technologies,” “social networks,” and “Facebook”) clustered in the search process to enhance the relationship between web-based communication technologies, isolation, and loneliness conditions of older people, as detailed in Figure 1. Facebook has been included in the set of keywords because, as specified in the introduction, this SNS is most used by older people. The search was conducted in English. We found 204 records, without duplicates, published from 2002 to 2019, that met the following eligibility criteria: (1) study uses experimental or quasi-experimental design; (2) study is based on pre-post controlled trials; (3) loneliness and/or well-being are outcomes; and (4) the target population is people aged over 60 years, considered the threshold for the aging process [26]. Two independent researchers (GC, DZ) screened the identified studies for their relevance based on title and abstract, and 185 studies were excluded: records not pertinent (n=69); qualitative studies (n=67); loneliness and well-being were not considered as the outcome (n=23); methodological papers (n= 6); literature reviews (n=13); and target population includes people under 60 years (n=7). A total of 19 articles met the inclusion criteria. Discrepancies between data extractions were resolved by team discussion and involving a third person (AG) as an arbitrator. After carefully reading the full texts of the articles, researchers excluded 8 more studies because they did not adopt experimental or quasi-experimental design. The selected articles were retrieved in full text and reevaluated by the authors for the final consensus on the inclusion. In the end, 11 papers were included in this review. No other references were identified by hand searching or analyzing the references of included articles.

### Data Extraction, Data Synthesis, and Analysis

In stage 4, we organized the materials to be analyzed. First, we ordered the collected papers by date from the oldest to the newest [27-37]. Moreover, we identified the review’s framework and related categories to provide our analysis (detailed in Table 1). Two macro areas of analysis have been identified: main characteristics of the protocols and main contents of the studies. For both of them, we identified a set of specific categories of analysis, one or more related main questions, and the items to collect. In stage 5, two researchers (GC, DZ) independently extracted the items based on the identified categories. To collect similar information on all studies, we performed a thematic and content analysis [38] based on the ex-post categorization of variables (eg, specific items) [39] to (1) detect the presence of variables in each selected study, (2) identify different modalities of selected variables (eg, tools used or different choices on sampling process), and (3) make them easy to read based on classification and summarization of specific contents. Last, to answer our research questions, the first author completed the data analysis using standardized self-made forms corresponding to Tables 2-4.

**Table 1:** Frameworks of analysis: general items, main questions, and detected items by analysis macro areas.

Categories and general items		Main questions	Detected items
**Protocol characteristics**			
	Keywords	What are the items of study declared by authors?	List of keywords
	Methods	Is the study experimental?	Presence of randomized trial study
	Sample	What is the dimension of the sample?	Number of participants in assessments
	Population	What are the age characteristics of population?	Range of age group Mean age
	Selection criteria	What are the applied selection criteria?	Place of living Previous experience with ICT^a^ use Healthy conditions Level of social engagement
	Recruitment	What is the recruitment strategy?	Place of recruitment
	Control group	How many control groups are included in the protocol?	Presence of control group Typologies of control groups
	Assessments	How many assessments are included?	Number and timing of follow-up evaluations
	Intervention	How long is the intervention? What activities are performed in the intervention?	Number of intervention weeks or years Presence of training course and training size
**Contents of studies**			
	Study issues	Is the study focused on SNS^b^ use?	Presence of SNS focus
	Outcomes	What are the outcomes included in the study? Is loneliness one of them?	List of outcomes Presence of loneliness as an outcome
	Evaluation tools	What are the evaluation tools used in the study? Which validated scales have been used?	Presence of validated scales Presence of self-made instruments List of validated scales
	Main results	What are the main results of study?	Summarize the results
	Declared limitations	What are the main limitations of study?	Summarize the limitation

^a^ICT: information and communication technology.

^b^SNS: social networking site.

**Table 2:** Keywords and protocol characteristics.

Author name and year	Keywords	Randomized trial	Sample size, baseline (f/u^a^)	Age range (mean)	Recruitment in care home	Control group	>1 f/u	Length of training (intervention)
White et al (2002) [27]	Older people; social isolation; internet; psychosocial impact	*^b^	100 (84)	59-83 (71.5)	*	P^c^	no	9 h (20 w)
Fokkema & Knipscheer (2007) [28]	N/A^d^	no	21 (12)	66+ (N/A)	no	V^e^	*	10 h (3 yrs)
Shapira et al (2007) [29]	Internet; senior well-being; personal sense of empowerment; Israel	no	46 (39)	70-93 (81.2)	*	A^f^	no	20 h (15 w)
Siegers et al (2008) [30]	Computer use; internet; well-being	*	236 (211)	64-75 (N/A)	no	P,P	*	4 h (54 w)
Woodward et al (2010) [31]	Gerontology; information and communication technologies; older adults; computer training; social support; mental health	*	83 (83)	60-89 (71.8)	no	P	*	24 h (24 w)
Blažun et al (2012) [32]	Older people; loneliness; computer training course; socialization; health; well-being	no	58 (45)	58-93 (72.9)	*	no	no	4 h (3 w)
Cotten et al (2012) [33]	Computers; internet; loneliness; social isolation; older adults; independent living; assistant living facilities	*	205 (205)	N/A (82.7)	*	A,P	*	N/A (8 w)
Myhre et al (2017) [34]	Executive functions; social interaction; social media; technology; training; working memory	*	43 (41)	75-86 (81.7 / 75.7?)	*	A,P	no	6 h (8 w)
Larsson et al (2016) [35]	Loneliness; social activities; social contact	*	30 (30)	61-89 (71.2)	no	P	*	N/A
Quinn (2018) [36]	Older adults; executive functions; social media training; experiment	*	34	N/A (76.5)	no	P	*	8 h (4 w)
Morton et al (2018) [37]	computers; internet; social connections; cognitive capacity; well-being	*	97 (76)	60-95 (80.7)	* not exclusive	P,D^g^	no	18 h (12 w)

^a^f/u: follow-up.

^a^*: yes, not available, or not declared in paper.

^a^P: passive control group or waiting list.

^a^N/A: not applicable.

^a^V: virtual control group by online survey.

^a^A: active control group.

^a^D: double intervention with different place of living and two control groups.

**Table 3:** Contents of studies: focus on social networking sites, outcomes, and tools.

Author name and year	Focus on SNS^a^	Loneliness	Beneficial effects on loneliness	Other outcomes	Specific questionnaire	Validated scales
White et al (2002) [27]	no	*^b^	no	Psychological and social well-being	no	UCLA Loneliness Scale, UCLAS^c^, CES-Depression^d^, PCLS^e^, CAS^f^
Fokkema and Knipscheer (2007) [28]	no	*	*	Distinction between social loneliness and emotional loneliness	no^h^	SJGLS-6^g^
Shapira et al (2007) [29]	no	*	*	Psychological well-being	no	UCLAS, DPFS^h^, DACL^i^, SAS^j^, PCS^k^, LSS^l^
Siegers et al (2008) [30]	no	*	no	Physical, social, and emotional well-being	no	SJGLS-6, SF-36^m^, SCL-90^n^, IADL scale^o^, EPQ-R^p^, ECS^q^
Woodward et al (2010) [31]	no	*	no	Mental health	*	Antonucci’s HMT^r^, Gagnè-MNSS^s^, CSE-16^t^
Blažun et al (2012) [32]	no	*	*	no	*	no
Cotten et al (2012) [33]	*	*	* weakly	Social well-being	*	RTLS-34^u^
Myhre et al (2017) [34]	*	*	no	Cognitive functions	no	UCLA Loneliness Scale, LSNS-18^v^, SPS-10^w^, RAVLT^x^, ReyCFT^y^, DSST^z^, DFRTT^aa^, TMT^bb^, COWAT^cc^, Miyake EFsT^dd^
Larsson et al (2016) [35]	*	*	*	no	no	UCLA Loneliness Scale, ESI^ee^, VAS^ff^
Quinn (2018) [36]	*	no	no	Cognitive functions	no	MMSE^gg^, COAST^hh^, SDMT^ii^, WAIS^jj^
Morton et al (2018) [37]	*	*	no	Computer attitude; sense of self-worth (competence, autonomy, and personal identity); cognitive and mental health	no	UCLA Loneliness Scale, SNAI^kk^, ACE-R^ll^, CES-D Depression^mm^, GAI-SF^nn^, GHQ-12^oo^, SWL^pp^, SWLS^qq^, CAS

^a^SNS: social networking site.

^b^*yes or not available.

^c^UCLAS: Revised UCLA Loneliness Scale.

^d^CES-Depression: CES-Depression Scale.

^e^PCLS: Perceived Control Life Situation.

^f^CAS: Computer Attitude Scale.

^g^SJGLS-6: 6-Item De Jong Gierveld Loneliness Scales.

^h^DPFS: Difficulties in Physical Functioning Scale.

^i^DACL: Depressive Adjective Checklist.

^j^SAS: Self-Anchoring Scale.

^k^PCS: Perceived Control scale.

^l^LSS: Life Satisfaction Scale.

^m^SF-36: 36-item Short Form Health Survey.

^n^SCL-90: 90-item Symptom Check List.

^o^IADL scale: Specific Questionnaire to Measure Daily Activities.

^p^EPQ-R: Subscales of the Eysenck Personality Questionnaire.

^q^ECS: External Control Scale.

^r^Antonucci’s HMT: Antonucci’s Hierarchical Mapping Technique.

^s^Gagnè-MNSS: Gagnè Motivation and Need Satisfaction Scale.

^t^CSE-16: Computer Self-Efficacy 16-item Scales.

^u^RTLS-34: Rasch Type Loneliness Scale 34-item.

^v^LSNS-18: Lubben Social Network Scale 18-item version.

^w^SPS-10: Social Provision Scale.

^x^RAVLT: Rey Auditory Verbal Learning test.

^y^ReyCFT: Rey Complex Figure Test.

^z^DSST: Digit Symbol Substitution Test.

^aa^DFRTT: Deary-Liewald Reaction Time Test.

^bb^TMT: Trail Making Test.

^cc^COWAT: Controlled Oral Word Association Test and Category Fluency Test.

^dd^Miyake EFsT: Miyake Executive Function Test.

^ee^ESI: Evaluation of Social Interaction.

^ff^VAS: visual analog scale.

^gg^MMSE: Mini-Mental State Examination.

^hh^COAST: California Older Adults Stroop Test.

^ii^SDMT: Symbol Digit Modalities Test.

^jj^WAIS: Wechsler Digit Span Forward and Backward subtest.

^kk^SNAI: Social Networking Activity Index.

^ll^ACE-R: Addenbrooke’s Cognitive Examination–Revised.

^mm^CES-D Depression: CES-D Depression Scale.

^nn^GAI-SF: Geriatric Anxiety Inventory Short Form.

^oo^GHQ-12: General Health Questionnaire.

^pp^SWL: 5-Item Satisfaction With Life Scale.

^qq^SWLS: The Satisfaction With Life Scale.

**Table 4:** Contents of studies: findings and limitations.

Author name and year	Results	Limitations
White et al (2002) [27]	Successful intervention. Improvement of PC use by all participants No significant correlation between self-reported health, activity limitations, and internet use	Short follow-up time to evaluate intervention effect in terms of loneliness Need for more intensive intervention Use of self-reported condition of PC use for selection criteria
Fokkema and Knipscheer (2007) [28]	Feeling of loneliness significantly decreased in both follow-ups (2 and 3 years after baseline) Greatest reduction took place in the first step (T0 to T1) Difference of reduction of loneliness between intervention group and control group is significant Emotional loneliness reduction is significant, unlike the results on social loneliness Qualitative findings confirm results that the internet helps to maintain contact with family or friends and it is a meaningful way to pass time	Social contacts of participants increased due to classes and tutoring included in the intervention. These could influence effects of loneliness Experiment was only performed once, and results should then be checked by repeat study Suboptimal matching of intervention and control groups created a problem with identifying the intervention as the reason for the reduction of loneliness
Shapira et al (2007) [29]	Positive effects on loneliness, less depression, more satisfaction with life, and more control and pleasure with their current quality of life	Short and simple intervention to analyze a complex phenomenon Language barrier in PC use Lack of analysis of secondary effects (eg, social environment and activism)
Siegers et al (2008) [30]	No significant differences (including for loneliness) were observed between groups and between times of follow-ups	Lack of analysis of changes in lifestyles Self-report measures of aspects of well-being Exposure time bias: 1 year too short to make structural changes but too long to measure small daily changes in attitude
Woodward et al (2010) [31]	No significant effect on loneliness and depressive symptoms between groups and between times Experimental group reported a significantly higher quality of life compared with control group No significant effect on social support outcome Significantly greater computer self-efficacy and use of ICTs^a^ for both groups	Randomization on people who agreed to participate made a self-selected sample not corresponding to a real population
Blažun et al (2012) [32]	Loneliness reduction was statistically significant, detected in pre-post analysis related to gender (female), living alone, living in town Improvement in independence of people living alone in town and their perception of safety	Different trials in country cases Data collection bias; questionnaire translation
Cotten et al (2012) [33]	Weak and negative correlation between going online and loneliness Moderate correlation between internet outcome variables and quality of communication Mean social relation network: 11.2 members—friends and family	Small sample Lack of analysis of detailed information on participants
Myhre et al (2017) [34]	Social outcome measured no significant change between pre- and posttimes	Small sample Randomization sample had been adapted by availability of individuals Socialization in group should have had an effect on results
Larsson et al (2013) [41]	Significant effect on satisfaction loneliness Online contacts were significantly improved No changes in offline contacts satisfaction	Lack of active control groups
Quinn (2018) [36]	No significant differences pre-post No significant improvement on MMSE^b^	Small sample Lack of detailed information on participants
Morton et al (2018) [37]	Significant cognitive improvements across time in the training but not control group This effect was mediated through a combination of increased social activity, improved self-competence, and preserved personal identity strength Indirect effects on mental health outcomes via these processes were also observed Larger improvement was detected in the residential care group (more socially restricted at the outset) No significant effect on satisfaction loneliness	Inability to disentangle the effects of training from the trainer visits High attrition rate Enduring relevance of the issues raised: importance of providing older adults with supportive training in the social use of internet technology might wane with current generations

^a^ICT: information and communication technology.

^b^MMSE: Mini-Mental State Examination.

## Results

### Protocol Characteristics: Methods

The main findings are summarized in Tables 2-4. Some methodological differences have been detected in study designs. First, 9 studies [27,29,31-37] were experimental with randomized sampling, while 2 [28,30] were quasi-experimental studies. In addition, 2 studies [27,34] were based on previous pilot studies. In the end, Larsson et al [35] used a crossover study and White et al [27] integrated the quantitative study with a short qualitative interview performed at the follow-up.

### Protocol Characteristics: Population, Sample, and Inclusion Criteria

A total of 953 participants were recruited for these studies, and 860 of them took part in the postintervention assessment. Thus, around 10% of participants dropped out of the experimental studies before the follow-up. The age groups were different in each of the analyzed studies: from the largest, 58 to 93 years, in the Blažun et al [32] and Morton et al [37] studies to the smallest, 64 to 75 years [29] and 75 to 86 years [32], defined by 11 years’ range (Table 2). The participants mean age, when declared, was over 71 years, with 4 studies ([29,33,34,37] reporting a mean age over 81 years.

Furthermore, the sample sizes were very heterogeneous among studies. Taking into consideration the follow-ups, most of the studies involved fewer than 50 participants [28,29,32,34-36], while in 3 studies, the sample size was more than 75 but less than 85 [27,31,37]. Only Siegers et al [30] and Cotten et al [33] proposed samples comprising more than 200 subjects. Power analysis to support the sample size definition was conducted in only one study [30].

The studies imposed a range of inclusion criteria (20 in total, see Multimedia Appendix 1). In the summarizing phase, we identified 4 categories of criteria: (1) health conditions, (2) level of experience on PC and ICT use, (3) place of residence, and (4) social engagement. Health condition was the most used inclusion criteria: only 4 studies did not provide health condition criteria to take part in the study [28,31,33,35]. Health was frequently conceptualized as cognitive ability. This information was collected as self-reported information by participants [28,34] or caregivers [27,32]. Four studies directly measured the cognitive level using the Mini-Mental State Examination , but heterogeneous thresholds are used to identify cognitively intact individuals: in the Morton et al [37] study 19 points were sufficient to be included in the sample, Siegers et al [30] fixed the minimum score at 24 points, and Blažun et al [32] and Myhre et al [34] put it at 26 points. In most of the selected studies, low level of knowledge and use of technologies has been used as a specific inclusion criterion. Some specific differences were detected: White et al [27], Fokkema and Knipscheer [28], and Morton et al [37] generally focused only on PC use, while Quinn [36] shifted the focus to SNS use. Myhre et al [34] considered both, underlining the complexity of social interaction based on use of technologies. Conversely, Siegers et al [30] and Larsson et al [35] selected people who were already experienced in using a PC. Last, the availability of a PC at home was required by Larsson et al [35] and Myhre et al [34], while Morton et al [37] required available space and infrastructure for internet use.

The place of residence criteria were strongly related to the recruitment process. Five studies [27,29,32-34] involved people in residential homes, communities, or day centers, but only Cotten et al [33] declared that living in residential homes was one of the selection criterion. Morton et al [37] included people living at home and in residential care to build a double comparison sample: intervention and control groups living in 2 different places. The remaining 5 studies recruited exclusively older people living at home.

Social engagement criteria were adopted in 2 studies: Larsson et al [35] selected retired people who reported loneliness and social isolation experiences, while Fokkema and Knipscheer [28] combined reported loneliness experiences with willingness to take part in the study.

### Protocol Characteristics: Control Groups, Follow-Up, and Interventions

All studies except for Blažun et al [32] benefited from at least one control group. The waiting list as a passive control group was a choice in 4 papers [27,31,35,36]. Fokkema and Knipscheer [28] chose to use an online survey as a virtual passive control group. Shapira et al [29] included only an active control group, with an alternative intervention to a selected part of the sample. Instead, to support the comparative control action, Myhre et al [34] and Cotten et al [33] combined active and passive control groups. Two passive control groups were used by Siegers et al [30] to compare the results between individuals not interested in attending the training course on PC use and those included in the waiting list despite their interest. Morton et al [37] identified 2 intervention groups and 2 waiting lists to further detect differences due to living places (at home or residential care institution).

Five protocols were characterized by a single follow-up at the end of the intervention [27,29,32,34,37]. In 2 cases, an additional follow-up was planned at 4 months [36] or 12 [30] months after the intervention. Fokkema and Knipscheer [28] proposed 2 long-term follow-ups at 14 months and 24 months after the baseline. Cotten et al [33] included multiple follow-ups at 3 months, 6 months, and 12 months. This choice is quite similar to what proposed by Woodward et al [31], based on 3 repeat measurements every 3 months after baseline (at 3 months, 6 months, and 9 months). In the crossover study by Larsson et al [35], participants were tested 2 times after baseline, as expected in this kind of method.

All experimental studies used training classes on PC or SNS as the main part of the intervention. In 4 studies, training did not last longer than 9 hours, and the intervention duration was often less than 8 weeks [32-34,36]. Most interventions included the provision of extra incentives to support ICT use (eg, tutoring and exercise sections [27-31,37]). Siegers et al [30] and Fokkema an Knipscheer [28] made a choice to invest in long-term online tutoring. Both offered short training (4 hours and 10 hours) before their long intervention. Siegers et al [30] preferred to use online tutoring, while in the study by Fokkema and Knipscheer [28] participants were supported and coached by visiting volunteers once every 2 or 3 weeks throughout the 3 years of the project. The protocol by Morton et al [37] provided 18 hours of training in the first month of intervention, while the second and third months were devoted to online tutoring.

### Contents of Studies: Focal Issues, Outcomes, and Tools Used

Papers published after 2013 considered SNS use an independent variable, while the previous ones focused their attention on PC use, including email activity (Table 3). Loneliness was analyzed as a specific well-being outcome by all studies except Quinn [36], who focused on self-perceived SNS impact on personal social life and personal relationship network without direct reference to loneliness. In particular, loneliness was the single outcome analyzed in Larsson et al [35] and Blažun et al [32]. Fokkema and Knipscheer [28], taking inspiration from Weiss’s theory, distinguished between social loneliness and emotional loneliness [40] and provided a measurement of both. Other studies presented additional outcomes focused on the health and well-being of older people: cognitive functions [32,34], cognitive and mental health [31,37], psychological effects [27,29,30], and emotional well-being [30]. The study by Morton et al [37] stressed sense of self-worth meant as autonomy, personal competence and personal identity. Social well-being (meant also as social isolation) was analyzed as participation in social activities [30] or social network ties and measured as quantity and quality of online communication [33,37].

International validated scales were the most commonly used measurement tools, but 2 studies included bespoke questionnaires created by the authors [32,33]. The 44 scales and/or tests were summarized in 5 categories: (1) aspects of social relationship life, (2) neuropsychological conditions, (3) clinical and physical well-being, (4) psychological well-being, and (5) ICT attitude and use. The complete list of validated scales with breakdown by category is provided in Multimedia Appendix 2.

Three studies [27,29,33] used the UCLA Loneliness Scale as a single perceived social measurement tool, while 2 studies combined that scale with others: Myhre et al [34] added the Lubben Social Network Scale 18-item and the Social Provisions Scale and Larsson et al [35] added the Evaluation of Social Interaction scale. Siegers et al [30] and Fokkema and Knipscheer [28] used the RTLS-34 scale exclusively. Woodward et al [31] combined the RTLS-34 with Antonucci’s Hierarchical Mapping Technique.

Clinical and physical well-being was investigated by Shapira et al [29] using the Difficulties in Physical Functioning Scale and by Siegers et al [30] using the Lawton Instrumental Activities of Daily Living Scale, 90-item Symptom Checklist ,and the 36-item Short Form Health Survey. Myhre et al [34], Larsson et al [35], and Quinn [36] included neuropsychological tests, measuring intelligence, attention, memory and executive function abilities (as detailed in Table 3). White et al [27], Morton et al [37], Woodward et al [31], Shapira et al [29], and Siegers et al [30] preferred to study the effects on the psychological well-being of older people using the Life Satisfaction Scale, depression level, and mastering of life phenomena. In addition, White et al [27], Morton et al [37], and Woodward et al [31] choose to use the Computer Attitude Scale and the 16-item Computer Self-Efficacy Scale.

### Contents of Studies: Findings and Declared Limitations

Five studies highlight the positive effect of ICT use including SNS use on the social relationships of older people (Table 4). The investigations by Shapira et al [29], Fokkema and Knipscheer [28], Blažun et al [32], and Woodward et al [31] highlight beneficial effects on loneliness and depressive symptoms, increased life satisfaction, and more control and pleasure with their current quality of life. One of them, Blažun et al [32], showed the reduction of loneliness stratified by gender: the improvement in perceived loneliness was more common among women than men. Conversely, Siegers et al [30], Morton et al [37], and Myhre et al [34] observed no statistically significant differences in pre-post evaluations, despite the data showing a decreasing trend of loneliness among participants. Even if Morton et al [37] underlined an improvement in both intervention groups included in their study, albeit one not statistically significant, the larger upgrade was detected in people living in residential care compared with those living at home. Fokkema and Knipscheer [28] stressed how the long-term (2 and 3 years after baseline) improvement on loneliness was statistically significant, unlike what had been detected at the first follow-up. However, participants in the training course of ICTs and SNSs improved their competence and sense of self-worth through increased social activity [37], but these positive effects on online communications do not impact personal satisfaction related to offline contacts [35]. Cotten et al [33] confirmed that the improvement of online activities did not influence the quality of communications or the size of the personal network in the social relationship: if, generally, the network comprised 11 people, most of them were old friends and family members. This assumption was further confirmed by the findings of Fokkema and Knipscheer [28]. The reduction of emotional loneliness proved significant, unlike the results on social loneliness. Moreover, the qualitative findings underline that, for the interviewed participants, the internet was a meaningful way to pass time because it helped maintain contact with family or friends.

In addition to general results on the main declared outcomes, some papers reported the effects of treatment on secondary outcomes in terms of the success of training [27]. Morton et al [37] identified additional indirect effects on mental health outcomes of older people involved in the study. In particular, increased personal competence on ICT use was related to improvement in the sense of self-worth, strength of personal identity, and self-esteem. Similarly, Blažun et al [32] detected an enhanced independence of participants living alone in town and in their perception of safety. The study by Quinn [36] detected the lack of statistical significance of SNS use on cognitive functions in older people.

The authors highlighted how their study protocols presented limitations. In 2 cases, the intervention duration was considered too short to assess ICT effect on older people’s loneliness [27,29]. White et al [27] and Siegers et al [30] underlined how an exposure time of less than 1 year was not enough to grasp a complex phenomenon as ICT effect on loneliness, but, at the same time, it is too long to measure small daily changes in older people’s attitudes. The sample size was identified as a focal limitation in 3 experimental studies [33,34,36]: small samples (composed of 30 or 50 people) do not seem able to monitor pre-post differences in the relationships between ICT use and older people’s emotional well-being, in particular loneliness. Also, Cotten et al [33] reported a similar limitation due to small sample size, despite the involvement of more than 205 individuals. Moreover, the issue of sample size should be linked to other limitations arising from the sampling process. In particular, the above mentioned authors [33,34,36] focused attention on the lack of detailed information about participant lifestyles, and, consequently, on the inability to perform integrative stratified analysis. Siegers et al [30] and Quinn [36] agreed with Cotten et al [33] about the lack of secondary analysis on participants’ lifestyle context (eg, social environment, attitude toward active lifestyle, or participation in volunteering associations). Instead, from the point of view of Myhre et al [34] and Woodward et al [31], the main limitation of their studies concerned the adaptation of the randomization process due to the availability of the individuals to participate in the training course. Two studies [27,30] reported limitations due to the chosen measures, in particular regarding the use of self-reported scales to assess loneliness. Last, some bias was observed due to the translation of research and intervention tools from the original language to participants’ language [29,32]. Some limitations coming from the structure of the interventions were identified by Fokkema and Knipscheer [28] and Morton et al [37]. In particular, the increase of social contacts between participants in the training course and their interactions with trainers and tutors could influence the effects on loneliness, although the study designs did not allow us to disentangle the effects of training from the trainer’s visits. In the end, Morton et al [37] placed the emphasis on how the issue of the impact of online communication in older age and on the benefits coming from specialized courses would be transcended, since new generations of older people will already be ICT users.

## Discussion

### Principal Findings

The analysis of the findings of the reviewed studies highlights the growing interest in assessing the impact of SNSs on the social well-being of elderly people. The existence of a positive effect of ICT use on older people’s loneliness seems to be confirmed, even if it is often a weak or not statistically significant effect. Moreover, the results underline how the use of ICTs has positive effects on the individual sense of self-worth, strength of personal identity, and self-esteem. The literature emphasizes the connection between these aspects of mental and psychological well-being and loneliness [42]. These results underline, once more, the hard work needed to analyze by experimental studies complex phenomena such as loneliness. The small number of experimental studies available in the literature on the effect of ICTs on social well-being and loneliness of older people and the limitations declared by the authors of the analyzed studies confirm this assumption. Indeed, many of these limitations concern the lack of control on the secondary variables that potentially influence the outcome, such as the effect of social contact during the training course, which is not easy to isolate [43]. In addition, the characteristics of lifestyle and level of social engagement of participants take on a specific relevance in the study designs. It is not by chance that the biggest limitation detected is small sample size, which does not ensure assessment of many of these secondary variables and, thus, a switch from specific findings to general assumptions on causal relationship. To confront the difficulty in defining the protocol, this review underlines how different methods are implemented to balance methodological accuracy and improvement of knowledge of the considered phenomenon. In addition, protocol designs were rarely supported by power analysis, pilot studies, or short qualitative studies [44].

Despite the low level of attrition in the selected studies (always below 10%), long- and medium-term studies involving older people need to take it into account carefully, because it could occur nonrandomly, thus posing challenges in the accuracy of results [45]. Furthermore, the heterogeneity of the selected population meets the debate on the definition of older people, conventionally defined as persons age 60 years. This definition is currently discussed in the literature [46]. Demographic trends and the literature underline how nowadays older age is setting in later [47]. This trend is confirmed by the sample composition: older people involved in the selected studies are generally aged 70 years or more.

In almost all of the reviewed studies, participants must have been early users of ICTs, but in some of them the availability of a PC is a fundamental participation criterion. This choice, although it alleviates the cost and management of the study, can represent a source of bias in the sampling process. Indeed, in almost all cases the ICT use is self-reported by participants and may correspond to a different level of actual knowledge and ability in using the PC.

This review stresses the strong impact of the chosen recruitment process on the target population and sampling design. In particular, recruitment by flyers mostly involves people living at home and with a high level of autonomy, while recruitment in residential homes could find less healthy people with some functional limitations (eg, hearing or walking) [48]. These conditions could influence the social life of participants significantly. The assessment of loneliness was often not considered as a prerequisite to be involved in the study, even if it was the main outcome of several studies. Considering that loneliness is a subjective feeling, its detection at baseline allows a pre-post measurement of individual change and a comparison between individuals with different starting conditions. The sample must ensure the availability of this measurement, but often the reviewed studies underlined a bias in the sampling process: people who volunteered to participate in the study were often active people with fewer loneliness experiences. The widespread inclusion of at least one control group supports the analysis of the causal relationship between dependent (health and well-being of older people) and independent (ICT use) variables compared with daily life activities (passive control group) or a different socialization intervention (active control group). The detected trend of single follow-up underlines how these studies are often aimed at evaluating the short-term effect of an intervention. To capture long-term effects, some studies proposed further follow-ups after 6 months or 12 months or, as done by Fokkema and Knipscheer [28], after 3 years. The choice of Fokkema and Knipscheer [28] seems to be successful because it detected a changing trend in significant loneliness, underlining how loneliness trends are better detected by long-term rather than short-time evaluation. However, this choice needs a longer intervention characterized by additional tutoring activities between follow-ups to support ICT use by participants. Otherwise, as declared by Fokkema and Knipscheer [28] and Morton et al [37], a long-term intervention could facilitate the interfering effect of internal social activities on the self-perception of loneliness.

In the last 5 years, in line with the broader use of SNSs, the number of studies that have observed the socializing role of ICTs has grown. Loneliness is the primary measured outcome of individual social well-being and is often considered strongly related to other well-being aspects, such as personal life satisfaction, cognitive health conditions, and the ability to manage daily life events. The heterogeneity and number of measurement tools used confirm the theoretical and methodological debate on how complex phenomena (such as loneliness or social isolation) should be measured [49]. The variety of validated scales ensures the appropriateness of measure for quantitative studies, but it does not allow us to read the inside aspects of a personal feeling of loneliness. The choice to use multiple scales, done by many authors, pushes us to a deeper understanding of the issue [50]. Moreover, the use of validating scales ensures reliability of measurements, but each scale measures a specific aspect of loneliness phenomenon, and the variety of scales between studies reduces the comparability of results.

### Limitations

Some limitations need to be considered in this review. First, the small amount of experimental articles in this area and their heterogeneity make it hard to produce a meta-analysis of the results and draw generalized conclusions. The decision to limit the literature search to articles published in English might have led to selection bias, although English is by far the leading language in this field of research. Second, the complex nature of the outcomes compared with the small size of the study samples limits the generalizability of the conclusions.

### Conclusions

This review aimed to clarify how experimental studies improve the understanding of the causal relationships between older people’s ICT use and their well-being concerning loneliness. Moreover, this review highlights the analysis of protocols applied to support the design of future research in this field. In particular, we compared 11 experimental and quasi-experimental studies published from 2002 to 2019. The characteristics of the analyzed study protocols (research questions, outcomes, evaluation tools, and treatments) highlighted difficulties in design, sampling, and management of the interventions. Nevertheless, despite the declared limitations, the overall findings are positive, highlighting the need for studying these issues with adequate methodological rigor. First, care is needed to discern the causal relationship between the dependent and independent variables from the effects of other intervening variables. Second, these difficulties affect the possibility of carrying out more than one follow-up—usually at the end of the intervention—and force the recruitment of small samples. The extensive use of the passive control group is an indirect effect of these difficulties. Moreover, this review underlines how the complex nature of loneliness and, even more, its relationship with ICT use, would require a complex and complete design of evidence-based studies, characterized by multivariable schemes and large sample sizes. On the other hand, randomized controlled studies allow the identification and analysis of causal relationships. The experimental studies included in the review show some difficulties and limitations in data collection due to the exclusive use of standardized tools to analyze the loneliness issue, in which individual feeling seems better detected by qualitative than quantitative methods.

The findings coming from the reviewed studies seem to confirm a beneficial effect—albeit weak—of ICT use on the well-being of older people in terms of reduced loneliness. The weakness of these results, along with the growing interest in the relationship between ICT use and loneliness in older age, draws attention to the need for development of further evidence-based studies. Future research in this field should take account of the need for studies with multidisciplinary design. The integration of clinical, psychological, and sociological research approaches would allow us to better verify primary and secondary outcomes of ICT use for older people’s well-being, including loneliness. Moreover, quantitative protocol studies could benefit from a larger randomized sampling—better if supported by power analysis—and by a short qualitative set of questions to improve the understanding and validity of the results.

## Appendix

Multimedia Appendix 1 Keywords and protocol characteristics.

Multimedia Appendix 2 Reference list of the international validated scales used in the reviewed studies.

## Abbreviations

ICT: information and communication technology
PRISMA: Preferred Reporting Items for Systematic Reviews and Meta-Analyses
SNS: social networking site
UCLA: University of California Los Angeles
