# Supplementary material for: The Effect of Information and Communication Technology and Social Networking Site Use on Older People’s Well-Being in Relation to Loneliness: Review of Experimental Studies
Source: J Med Internet Res. 2021 Mar 1;23(3):e23588. doi: 10.2196/23588 (PMC7961406; doi:10.2196/23588)
Supplement: Multimedia Appendix 1 [file jmir_v23i3e23588_app1.docx]

Multimedia Appendix 1. Keywords and protocol characteristics.

|  | *Keywords* | *Randomized trial* | *SampleUnits*  *(Baseline; follow-up)* | | *Age group*  *(range)* | *Mean Age (years)* | *Selection criteria* | *Recruitment in retirement/care home* | *Control group* | *>1 follow-up* | *Training size (h)/*  *Intervention size (weeks or years)* |
| --- | --- | --- | --- | --- | --- | --- | --- | --- | --- | --- | --- |
| *a.1*  *White et al. (2002)* [29] | Older people, social isolation,  Internet, psychosocial impact | * | 100  84 | | 59-83  (24 ) | 71.5 | B.1, C.2, C.5 | * | P | º | 9h  20 weeks |
| *a.2*  *Fokkema & Knipscheer (2007)* [30] | n.a | º | 21  12 | | 66+ | n.a | A.1, B.1, B.5, C.1 C.6 C.7 D.2 D.1 | º | V | * | 10h  3 years |
| *a.3*  *Shapira et al. (2007)*  [31] | Internet; senior’s well-being; Personal sense of empowerment; Israel | º | 46  39 | | 70 -93  (23 ) | *81.2* | º | * | A | º | 20h  15 weeks |
| *a.4*  *Siegers et al. (2008)* [32] | Computer use; Internet; Well-being | * | 236  211  204 | | 64 -75  (11 ) | n.a | B.4, C.3 | º | P, P | * | 4h  54 weeks |
| *a.5*  *Woodward et al. (2010)* [33] | Gerontology; Information and communication technologies (ICTs) ; Older adults; Computer training; Social support; Mental health; | * | 83  83 | | 60-89  (29 ) | *71.8* | A.1 | º | P | * | 24h  24 weeks |
| *a.6*  *Blažun et al. (2012)*  [34] | Older people, Loneliness; Computer training course, Socialisation; Health; Well-being | º | 58  45 | | 58 – 93  (35 ) | *72.9* | C.2; C.5 | * | º | - º | 4h  3 weeks |
| *a.7*  *Cotten et al. (2012)* [35] | Computers; Internet; Loneliness; Social isolation; Older adults;  Independent living; Assistant living facilities. | *** | 205  205 | | n.a | *82.7* | A.2 | * | A  P | * | n.a.  8 weeks |
| *a.8*  *Myhre et al. (2017)* [36] | Executive functions, Social interaction; Social media; Technology; Training; working memory | *** | 43  41 | | 75 -86  (11 ) | 81.7  75.7 | B.1; B2; B.3 C.1, C.2 C.4 | * | A  P | º | 6h  8 weeks |
| *a.9*  *Larsson et al. (2016)*[37] | Loneliness; Social activities;  Social contact; | * | 30  30 | | 61 -89  (28 ) | *71.2* | A.1;B.3; B.4, D.2, D.3 | º | P | * | n.a. |
| *a.10*  *Quinn K. (2018)* [38] | Older adults; Executive functions;  Social media training; Experiment | * | 34 | | n.a | 76.5 | C.3, B.2; A.1 | º | P | * | 8h  4 weeks |
| *a.11*  *Morton et al.*  *(2018)* [39] | computers, Internet, social connections, cognitive capacity, well-being | * | 97  76 | | 60-95  (35 ) | 80.7 | A.1,A.2, C.2, C.8, C.9, B.1, B.6 | *  Not exclusive | P  D | º | 18h  12weeks |
| **Legend**  º not, * yes, n.a. not available/not declared in paper.  *selection criteria:*  A: living at home (A.1) or residential homes (A.2)  B: early user of PC (B.1) or SNSs (B.2); had a PC (B.3), PC user (B.4); no negative attitude toward computers (B.5); available space and infrastructure for Onternet use (B.6) | | | | C: healthy condition: self-report (C.1); Cognitive intact (C.2); MMSE ≥24(C.3); MMSE ≥26(C.4); Identified by caregivers or professionals in residential care (C.5);ability to work with a standard PC (C.6); sufficient sight and hearing abilities (C.7); six-item screener (Callahan et al,2002) (C.8); MMSE ≥19(C.9)  D: social engagement: Willingness to take part in the study. (D.1); reporting loneliness and social isolation experiences (D.2); Retired (D.3)  *Control group:* Active control groups (A); Passive Control group or waiting list (P); Virtual Control group by online survey (V); double intervention with different place of living and two control groups (D) | | | | | | | |
