# Supplementary material for: The Effect of Information and Communication Technology and Social Networking Site Use on Older People’s Well-Being in Relation to Loneliness: Review of Experimental Studies
Source: J Med Internet Res. 2021 Mar 1;23(3):e23588. doi: 10.2196/23588 (PMC7961406; doi:10.2196/23588)
Supplement: Multimedia Appendix 2 [file jmir_v23i3e23588_app2.docx]

**Multimedia Appendix 2: Reference list of the international validated scales used in the reviewed studies**

*Social relationship life’s aspects*

- UCLA loneliness Scale [1]
  - UCLA Loneliness scale [2]
  - Revised UCLA Loneliness scale (UCLAS) [3]
  - Lubben Social Network Scale 18-item version (LSNS-18) [4]
  - Social Provision Scale (SPS-10) [5]
  - Evaluation of Social Interaction (ESI) [6]
  - Rasch Type Loneliness Scale 34 items (RTLS-34) [7]
  - 6-Item De Jong Gierveld Loneliness Scales (SJGLS-6) [8]
  - Social networking activity index (SNAI) [9]
  - Antoniucci’s hierarchical mapping technique [10]

*Neuropsychological conditions*

- Mini-Mental State Examination (MMSE) [11]
- Rey Auditory Verbal Learning test [12]
- T.10-Rey Complex Figure Test (ReyCFT) [13]
- Digit Symbol Substitution Test (DSST) [14]
- Deary-Liewald Reaction Time Test (DLRTT) [15]
- Trail Making Test (TMT) [16,17]
- Controlled Oral Word Association Test and Category Fluency Test (COWAT) [18]
- Miyake executive function test (Miyake EFsT) [19]
- Visual Analogue Scales (VAS) [20]
- California Older Adults Stroop Test (COAST) [21]
- Symbol digit modalities test (SDMT) [22]
- Wechsler Digit Span Forward and Backward subtest (WAIS) [23]
- The Addenbrooke’s Cognitive Examination revised (ACE-R) [24]

*Clinical and physical well-being*

- Difficulties in physical functioning scale (DPFS) [25]
- 36-items Short Form Health Survey (SF-36) [26]
- 90-items Synthom Check List (SCL-90) [27]
- Specific questionnaire to measure daily activities (IADL scale ) [28]

*Psychological well-being*

- Depressive adjective checklist (DACL) [29,30]
- Self-anchoring scale (SAS) [31]
- Perceived control Scale (PCS) [32]
- Subscales of the Eysenck Personality Questionnaire(EPQ-R) [33]
- Life satisfaction scale (LSS) [34]
- External Control Scale(ECS) [35]
- CES-depression scale [36]
- Perceived Control life situation (PCLS) [37]
- CES- D depression scale [38]
- Geriatric Anxiety Inventory Short Form (GAI-SF) [39]
- General Health Questionnaire (GHQ-12) [40]
- Five Items Satisfaction With Life Scale (SWL) [41]
- Satisfaction questionnaire The Satisfaction With Life Scale (SWLS) [42]
- Gagnè Motivation and Need Satisfaction Scale (Gagnè-MNSS) [43]
- Geriatric depression scale (GDS) [44]

*ICT use*

- Computer Attitude Scale (CAS) [45]
- Computer self-efficacy 16 items scales (CSE-16) [46]

References

1. Russell D, Peplau LA, Cutrona CE. The revised UCLA Loneliness Scale: Concurrent and discriminant validity evidence. J Pers Soc Psychol 1980; [doi: 10.1037/0022- 3514.39.3.472]
2. Russell DW. UCLA Loneliness Scale (Version 3): Reliability, validity, and factor structure. J Pers Assess 1996; [doi: 10.1207/s15327752jpa6601_2]
3. Russell D, Cutrona CE, Rose J, Yurko K. Social and emotional loneliness: An examination of Weiss’s typology of loneliness. J Pers Soc Psychol 1984; PMID:6737214
4. Lubben J, Gironda M. Measuring social networks and assessing their benefits. Soc Networks Soc Exclusion Sociol Policy Perspect 2017.
5. Cutrona CE, Russell DW. The Provisions of Social Relationships and Adaptation to Stress. Adv Pers Relationships 1987;1:37–67. Available from: https[://w](http://www.researchgate.net/publication/271507385)ww[.researchgate.net/publication/271507385](http://www.researchgate.net/publication/271507385)
6. Griswold LA, Townsend S. Assessing the sensitivity of the evaluation of social interaction: Comparing social skills in children with and without disabilities. Am J Occup Ther 2012; [doi: 10.5014/ajot.2012.004051]
7. de Jong-Gierveld J, Kamphuls F. The Development of a Rasch-Type Loneliness Scale. Appl Psychol Meas 1985; [doi: 10.1177/014662168500900307]
8. Gierveld JDJ, Van Tilburg T. A 6-item scale for overall, emotional, and social loneliness: Confirmatory tests on survey data. Res Aging 2006; [doi: 10.1177/0164027506289723]
9. Cohen S, Doyle WJ, Skoner DP, Rabin BS, Gwaltney JM. Social ties and susceptibility to the common cold. J Am Med Assoc 1997; PMID:9200634
10. Antonucci TC. Measuring social support networks: Hierarchial mapping technique. Gener J Am Soc Aging 1986; PMID:25246403
11. Folstein MF, Robins LN, Helzer JE. The Mini-Mental State Examination. Arch Gen Psychiatry. 1983. PMID:6860082
12. Schmidt M. Rey auditory verbal learning test: A handbook. Los Angeles: CA: Western Psychological Services.; 1996. Available from: <http://v-psyche.com/doc/Clinical> Test/Rey Auditory Verbal Learning Test.docx
13. Meyers JE, Meyers KR. Rey Complex Figure Test under Four Different Administration Procedures. Clin Neuropsychol 1995; [doi: 10.1080/13854049508402059]
14. Lezak MD, Howieson DB, Loring DW, Hannay JH, Fischer JS. Neuropsychological Assessment. Oxford University Press. New York 2004;
15. Deary IJ, Liewald D, Nissan J. A free, easy-to-use, computer-based simple and four- choice reaction time programme: The Deary-Liewald reaction time task. Behav Res Methods 2011; PMID:21287123
16. Horton AM, Hartlage LC. The Halstead-Reitan neuropsychology test battery: Theory and clinical interpretation second edition. Arch Clin Neuropsychol 1994; [doi: 10.1093/arclin/9.3.289]
17. Corrigan JD, Hinkeldey NS. Relationships between Parts A and B of the Trail Making Test. J Clin Psychol 1987; [doi: 10.1002/1097-4679(198707)43:4<402::AID- JCLP2270430411>3.0.CO;2-E]
18. Benton A, deS K, Sivan A. Multilingual aphasia examination. 1994;
19. Miyake A, Emerson MJ, Friedman NP. Assessment of executive functions in clinical settings: Problems and recommendations. Semin Speech Lang 2000; PMID:10879548
20. Larsson J, Björkdahl A, Esbjörnsson E, Sunnerhagen KS. Factors affecting participation after traumatic brain injury. J Rehabil Med 2013; PMID:24002312
21. Pachana NA, Thompson LW, Marcopulos BA, Yoash-Gantz R. California older adult stroop test (Coast): Development of a stroop test adapted for geriatric populations. Clin Gerontol 2004; [doi: 10.1300/J018v27n03_02]
22. Smith A. Symbol digit modalities test: Manual: Western Psychological Corporation. 2002;
23. Wechsler D. Wechsler memory scale - Third edition administration and scoring manual. San Antonio, TX Psychol Corp 1997;
24. Mioshi E, Dawson K, Mitchell J, Arnold R, Hodges JR. The Addenbrooke’s Cognitive Examination revised (ACE-R): A brief cognitive test battery for dementia screening. Int J Geriatr Psychiatry 2006; [doi: 10.1002/gps.1610]
25. Zung WWK. A Self-Rating Depression Scale. Arch Gen Psychiatry 1965; [doi: 10.1001/archpsyc.1965.01720310065008]
26. Ware, Jr. JE, Gandek B. The SF-36 Health Survey: Development and Use in Mental Health Research and the IQOLA Project. Int J Ment Health 1994; [doi: 10.1080/00207411.1994.11449283]
27. Arrindel W, Ettema J. Handleiding bij een multidimensionele psychopathologie- indicator. Symptom Checklist SCL-90.(Herziene uitgave).[Manual for a multidimensional. 2003;
28. Graf, C. The Lawton instrumental activities of daily living (IADL) scale. MedSurg Nursing 2008, 17(5), 343-345.
29. Lubin J, Tate J. Formal moduli for one-parameter formal Lie groups. Bull la Soci&#233;t&#233; math&#233;matique Fr 1966; [doi: 10.24033/bsmf.1633]
30. Lubin B, Collins JF, Seever M, Whitlock R V. Readability of the depression adjective check lists (DACL) and the multiple affect adjective check list‐revised (MAACL‐R). J Clin Psychol 1991; [doi: 10.1002/1097-4679(199101)47:1<91::AID- JCLP2270470114>3.0.CO;2-#]
31. Cantril H. Pattern of human concerns. 1965; Available from: <http://agris.fao.org/agris-> search/search.do?recordID=US201300321344
32. Pearlin LI, Schooler C. The structure of coping. J Health Soc Behav 1978; PMID:649936
33. Eysenck H. Manual of the Eysenck Personality Questionnaire (adult and junior) 1975. Hodder and Stoughton, London.
34. Back K, aging CG-S aspects of, 1966 undefined. Retirement and self-ratings. Duke Univ Press Durham, NC.
35. Andriessen JHTH. Interne of externe beheersing. Ned Tijdschr voor Psychol 1972;2(7):173–199.
36. Kohout FJ, Berkman LF, Evans DA, Cornoni-Huntley J. Two Shorter Forms of the CES-D Depression Symptoms Index. J Aging Health 1993; PMID:10125443
37. Eizenman DR, Nesselroade JR, Featherman DL, Rowe JW. Intraindividual variability in perceived control in an older sample: The MacArthur successful aging studies. Psychol Aging 1997; PMID:9308096
38. Radloff LS. The CES-D Scale: A Self-Report Depression Scale for Research in the General Population. Appl Psychol Meas 1977; [doi: 10.1177/014662167700100306]
39. Byrne GJ, Pachana NA. Development and validation of a short form of the Geriatric Anxiety Inventory - The GAI-SF. Int Psychogeriatrics 2011; [doi: 10.1017/S1041610210001237]
40. Goldberg D, Williams P. General Health Questionnaire (GHQ). Swindon, Wiltshire, UK: nferNelson; 2000. Available from: <http://www.bsw.ugent.be/VVGP/structuurfiche_GHQ.pdf>
41. Diener E, Emmons RA, Larsem RJ, Griffin S. The Satisfaction With Life Scale. J Pers Assess 1985; PMID:16367493
42. Diener E, Emmons RA, Larsem RJ, Griffin S. The Satisfaction With Life Scale. J Pers Assess 1985; PMID:16367493
43. Gagné M, Ryan RM, Bargmann K. Autonomy Support and Need Satisfaction in the Motivation and Well-Being of Gymnasts. J Appl Sport Psychol 2003; [doi: 10.1080/714044203]
44. Yesavage JA, Brink TL, Rose TL, Lum O, Huang V, Adey M, Leirer VO. Development and validation of a geriatric depression screening scale: A preliminary report. J Psychiatr Res 1982; PMID:7183759
45. Jay GM, Willis SL. Influence of direct computer experience on older adults’ attitudes toward computers. Journals Gerontol 1992; [doi: 10.1093/geronj/47.4.P250]
46. Murphy CA, Coover D, Owen S V. Development and Validation of the Computer Self- Efficacy Scale. Educ Psychol Meas 1989; [doi: 10.1177/001316448904900412]
